# PicPsy: A new bank of 106 photographs and line drawings with written naming norms for Spanish-speaking children and adults

**DOI:** 10.1371/journal.pone.0238976

**Published:** 2020-09-14

**Authors:** Naroa Martínez, Helena Matute, Edurne Goikoetxea

**Affiliations:** Departamento de Fundamentos y Métodos de la Psicología, Universidad de Deusto, Bilbao, Spain; Italian National Research Council, ITALY

## Abstract

The use of pictures as experimental stimuli is a frequent practice in psychological and educational research. In addition, picture-naming task allows the study of different cognitive processes such as perception, attention, memory and language. Line drawings have been widely used in research to date but it has begun to be highlighted the need for more ecological stimuli such as photographs. However, normative data of a photographic set has not been published yet for use with children. We present PicPsy, a new standardized bank of photographs and matched line drawing. We collected written picture-naming norms for name agreement, unknown responses, alternative names, familiarity and visual complexity. A total of 118 native Spanish-speaking children in grades 3–4 participated in the study. For comparison purposes, 89 adults were also included in the study. Child and adult performance was highly correlated, but we found significant age group differences in all variables examined except for visual complexity. Researchers and teachers could benefit from using the new standardized bank reported here which is published under public domain license. The data and materials for this research are available at the Open Science Framework, https://osf.io/nyf3t/.

## Introduction

The use and manipulation of pictures as experimental stimulus is common practice in psychological and educational research with children and adults. In picture naming tasks, using well-controlled stimuli is crucial since different variables may influence cognitive processing. For instance, some of the variables that increase name agreement (i.e., an indicator of consensus on the naming of a picture in a sample) and reduce name latencies are: High frequency and familiarity of the word represented by the picture, an early age of acquisition of the word represented, and low visual complexity of the picture (e.g. [1–3] for studies with adults; [4] for a study with children). Also, in picture naming research there are some variables that are related to a conceptual approach (such as name agreement, familiarity and imageability) and other variables that examine picture naming as a visual representation (such as visual complexity and image agreement; [5]). Moreover, previous studies with adults have examined how certain characteristics of visual representations influence recognition. For example, there is evidence that naming performance varies as a function of picture type, that is, when the picture is a line drawing or a photograph. It has been showed that there are shorter naming latencies, higher naming accuracy [6, 7], and different sources of name disagreement [8] for photographs than for line drawings. Similarly, in a recent comparison of photographs versus line drawings, Martínez and Matute [9] showed that children produce better name agreement indices, give fewer incorrect alternative names, and rate as visually more complex the images represented in photographs than in line drawings. Moreover, some researchers have suggested the existence of differences in perceptual processing of photographs versus line drawing, such as, for instance, stronger embodiment representations for photographs of manipulable objects as compared to line drawings [10].

In order to allow for an appropriate experimental control of the influence of the stimuli on task execution, normative studies are necessary. They provide information about the variables of fundamental relevance to cognitive processing in picture naming tasks [3, 4]. However, and as we will show below, there is a surprising lack of pictures normative studies in children, particularly if we take into account that pictures are commonly used with Primary School children. For example, we are concerned that the only picture naming norms in Spanish-speaking children that we are aware of are available in Cuban Spanish [11]. Therefore, attending to the intercultural differences, and given that Spanish is one of the most frequently used languages in the world, we believe it is important to develop a normative picture naming bank for Castilian Spanish-speaking children. In addition, previous research has focused in line-drawing stimuli, but nowadays the availability of high definition photographs as experimental stimuli is increasing and, as mentioned above, they cannot be used interchangeably [9]. However, as far as we know, no photograph norms from children has been published. Photograph norms from adult samples are available, but adults´ data can hardly be generalized to children samples [4, 12, 13]. The present research presents our contribution to this goal by presenting normative data from line drawings and photographs of Spanish-speaking children. These norms and picture bank are freely available and open to any researchers and educators that may need them. In what follows, we first elaborate further on the need to present this particular set of norms, and then we will describe our methods, stimuli, and norms. Given the possible confusion that can be generated by the terms picture and naming, in this article we will refer to picture as the visual stimuli representing a concept and naming will be used to refer to the task that requires assigning a word (a lexical and semantical representation) to a visual stimuli.

### Line-drawing standardized sets

Line drawings are schematic, simple and prototypical representations of concepts that have been widely used in research with adults since the publication of the pioneering study of Snodgrass and Vanderwart [3] to date. This standardized picture database consists in 260 black-and-white line drawings with norms for a series of variables, such as name agreement, image agreement, familiarity and visual complexity. Since then, this set continues to be relevant in basic and applied research and has been adapted to numerous languages, such as, for instance, Chinese [14], French [15], Dutch [16], Italian [17], Icelandic [18], Japanese [19], and Russian [20]. Likewise, it has also been adapted to different linguistic and sociocultural contexts. For instance, in Spanish-speaking countries, it has been used in Mexico [21], Cuba [22], Argentina [23, 24], and Spain [25–27].

Currently, there are also other banks of line drawings with normative data (e.g., [28, 29]), and in different languages. For instance, the International Picture-Naming Project (IPNP [30], in English, German, Italian, Bulgarian, Hungarian, Mandarin and Mexican Spanish), the Protocole Européen de Dénomination Orale d'Images (PEDOI [31, 32], in Dutch, English, German, French, Italian, Russian, Swedish and Spanish) and the Multilingual Picture databank (MultiPic [33], in Spanish, British English, German, Italian, French and Dutch).

Most of the research has been carried out with adults; however, some normative studies were carried out with children of different age ranges. To our knowledge, all of them used the original set of Snodgrass and Vanderwart [3]. It has been adapted to English ([34], with norms of 40 children from 7 to 9 years old), Chinese ([35], with norms of 66 children from 4 to 6 years old); French ([12], with norms of 1,440 children from 3 to 8 years old); and Cuban Spanish ([11], with norms of 150 children from 5 to 12 years old). In addition, we are only aware of two studies that expand the Snodgrass and Vanderwart [3] set with norms for children. One of them is the set published by Cycowicz et al. [4], which included 400 line drawings with norms of 30 English-speaking children from 5 to 6 years, as well as norms of 36 Portuguese-speaking children from 5 to 7 years old [13]. The other one is the International Picture-Naming Project [30, 36] which included norms of 250 drawings of 34 Italian-speaking children from 5 to 6 years old [37]. At present, available normative datasets of line drawings are often composed by pixelated stimuli (e.g. [3, 4, 30]) and these result are of poor quality compared to the images that we are all used to find in our daily life.

Finally, several studies have reported differences across languages and cultures in adults [30, 31], while this issue has been rarely studied with children. One study examining these differences in children was conducted by Wang et al. [35]. Their results showed significant differences in *H* index, familiarity and image complexity between Chinese and American children and in the expected name agreement between Chinese and French children. According to the authors, these results may reflect differences in language but they also indicate cross-cultural differences in the image properties in children from different cultures. The researchers highlighted the need for culturally specific norms when using pictorial stimuli with children.

### Children and adults comparisons in line-drawing naming

Comparison research in line-drawing naming indicates that there are differences between children and adults in name agreement, modal name assigned (i.e., the most common name given to a picture in a sample), number and variability of alternative names provided, and percentage of unknown responses [4, 13, 34]. For example, in English, the seminal work offered by Cycowicz et al. [4] revealed that children provided modal names that differed from adults’ modal names for 13.5% of the pictures and produced different and a larger number of alternative names than adults did. Moreover, children reported not knowing the name or object for 9.8% of the pictures while the adults reported not knowing only 1.7% of the pictures. Previous studies also found that children were slower than adults in picture naming [37]. For example, D'Amico et al. [37] revealed that, in Italian, adults´ naming mean overall reaction time to produce the target name was 950 ms, 300 ms faster than children. The ratings of familiarity and visual complexity in previous literature were similar (though slightly lower) in children than adults [4, 13, 34].

Some researchers have pointed out that although the name agreement measures and naming latencies varied in children and adults, these differences are trivial, especially in older children, and the performance correlated highly in both age groups [34, 37]. However, we should be cautious with these conclusions because few studies made comparisons directly between children and adults using the same set of stimuli for both age groups, and they were carried out with small samples (< 40 children; [13, 37]). Furthermore, apart from quantitative differences, Cannard, Blaye, Scheuner, & Bonthoux [38] highlighted the importance of qualitative differences in normative research when the participants are children. One reason is that some modal names provided by the children differed from the names expected and provided by adults and, indeed, children present numerous mistakes. For instance, Pompéia et al. [13]compared qualitative differences in modal names and found that Portuguese-speaking children named 103 concepts (around 25%) differently from adults, and most of those were mistakes (e.g., *cockroach* for *beetle*). These results were in line with those provided by Cycowitz et al. [4]. The qualitative analysis of object names also makes it possible to identify the comprehension of word meanings of children from the earliest usages and it has been observed that the extensions' of children's objects name are like those of adults [39], but children are more likely than adults to omit responses because of lack of knowledge. Consequently, authors such as Cannard et al. [38] emphasize that previous literature comparing naming responses across age groups has underestimated the importance of these qualitative differences and warn about the need to be cautious when using the measures of picture naming in developmental studies, because they are dependent on modal name. In addition, the qualitative study of responses contributes to explore different cognitive processes involved in picture naming such as perceptual processing, activation of semantic information, lexical selection or name retrieval [40, 41].

### Photograph standardized sets

Traditionally, research has focused on line drawings, but the standardization of photographic stimuli is progressively increasing in recent years because digital photography is today easily accessible due to technological development.

Photograph stimuli are realistic representations of objects, as they include details such as texture, shades, brightness, and volume cues. Thus, the interest on more ecological stimuli than drawings has begun to be highlighted [42, 43]. To our knowledge, the largest existing normative photograph dataset is the Bank of Standardized Stimuli (BOSS; [44, 45]). It contains 1468 photographs with norms collected from English- and French- speaking adults. Other photographic datasets with norms for adults are the set published by Viggiano et al. [43], in English and Italian; the Hatfield Image Test [46], in English; the C.A.R.E. set [47] in English; the set of Shao & Stiegert [48] in Dutch; the set of Saryazdi, Bannon, Rodrigues, Klammer, & Chambers [49], in Turkish. The ecological version of the Snodgrass and Vanderwart [3] set [42] is the only one in Spanish, and has recently been published also in Italian [50].

To the best of our knowledge, no standardized photographic set with norms for children has been published, despite its potential use as well-controlled stimuli in picture naming tasks in Primary School. In particular, colored photographs offer a rich representation of real objects, are more ecological than line drawings, and their use is increasingly widespread.

Additionally, although the number of online websites that offer free photographs in the public domain have increased (see publicdomainpictures.net for a website of public domain pictures), very few standardized banks are easily accessible with a license description and fewer are in the public domain (e.g. [47, 48], for normative picture sets under a CC0 license). If standardized picture banks were published under a public domain license, their use could be extended to assessment and educational intervention materials, given that free and unrestricted use of controlled stimuli would be allowed, even in commercial tests and textbooks.

#### Spoken and written picture naming sets

Normative studies presenting standardized sets of pictures have been carried out with both the spoken naming task (e.g., [3, 30, 32, 43, 46]) and the written naming task (e.g., [2, 27, 28, 33, 44, 45, 47, 49, 50]). Some studies with children have also carried out the picture naming task in a written form [9, 34]. Nevertheless, these written naming studies are limited to those that included older children, thus being able to ensure that participants had some minimal orthographic skills to carry out the written task.

It has been noted that research on written picture naming is psychologically interesting, but it has not been researched enough [27]. On the one hand, written picture naming is of great relevance for the understanding of language production since picture naming has been widely used to explore the cognitive processes involved in word production (e.g., [51–54]). Although both spoken and written naming enables collecting measures of name agreement, written naming also allows collecting additional information such as spelling agreement (i.e., indicator of consensus on the spelling given to the name of a picture in a sample; e.g., [27]) and the time course of production within-word writing [2, 55, 56]. On the other hand, picture naming in written form allows both, group assessment and self-administration, making it easy to arrange the task in electronic format [28], which substantially reduces the time of data collection. This allows the sample to be larger and can facilitate expansion to other regions or countries via the internet [28]. When conducting normative studies with children, spoken naming is very laborious and time-consuming, so studies usually have the methodological flaw of analyzing data derived from very small samples. For example, in the seminal study by Cycowitz et al. [4] the sample was comprised of only 30 children (21 in kindergarten, 7 in first grade and 2 in second grade). Written naming offers the possibility of performing the tasks collectively, substantially reducing administration time and allowing the evaluation of a larger number of children.

Although the previous literature does not usually differentiate between the sets collected orally and those in written form, some studies have examined possible differences in picture naming effects between both strategies. According to these studies, written picture naming shows many of the same effects (name agreement, age of acquisition, semantic interference, or image variability) as spoken naming [27, 55, 57]. In a recent work, Torrance et al. [27] provides a dataset of written picture naming in 14 different languages. They found similar effects in written picture naming as those found in spoken picture naming. These results were confirmed for the variety of languages. In addition, the name agreement was roughly similar between written and comparable spoken datasets, but the authors suggest that more data are needed to clarify whether the name agreement might differ between spoken and written production. To summarize, there is a lack of standardized photograph banks for children, a population that requires well-controlled visual stimuli and one that could benefit the most from the use of high-quality ecological pictures. In addition, few normative picture sets are freely available with a license description. The aim of the present research was to provide a new bank of photographs and matched line drawings in the public domain with norms of several psycholinguistic variables in a sample of at least 100 Spanish-speaking children. For comparison purposes, adults were also included in the study using the same stimuli and tasks for both age groups. Unlike previous studies but as recommended by some authors [38], we had included in the analysis the measures of name agreement and the analysis of omissions and errors.

## Material and methods

### Participants

A total of 118 Spanish-speaking children from 3rd and 4th grade of Primary School (52% female, *M* age = 8 years 9 months, *SD* = 0.6, *range =* 7 to 10 years) and 89 adults (79% female, *M* age = 23 years 10 months, *SD* = 8.3, *range =* 18 to 58 years) participated in the study. All had Spanish as a mother tongue and none of them had received a diagnosis of neurological damage, or speech or language problems.

All of the children attended public schools in different regions of Northern Spain (Cantabria, *n* = 80; and Burgos, *n* = 38). Adults were mostly undergraduate students from Northern Spain as well (Vizcaya).

### Ethics statement

The ethics committee of the University of Deusto approved the procedure used in the present study (Ref: ETK-14/17-18). Written informed consent was collected from the parents or guardians of the children included in the study. Adults gave their informed consent to participate.

### Stimuli

**Fig 1:**
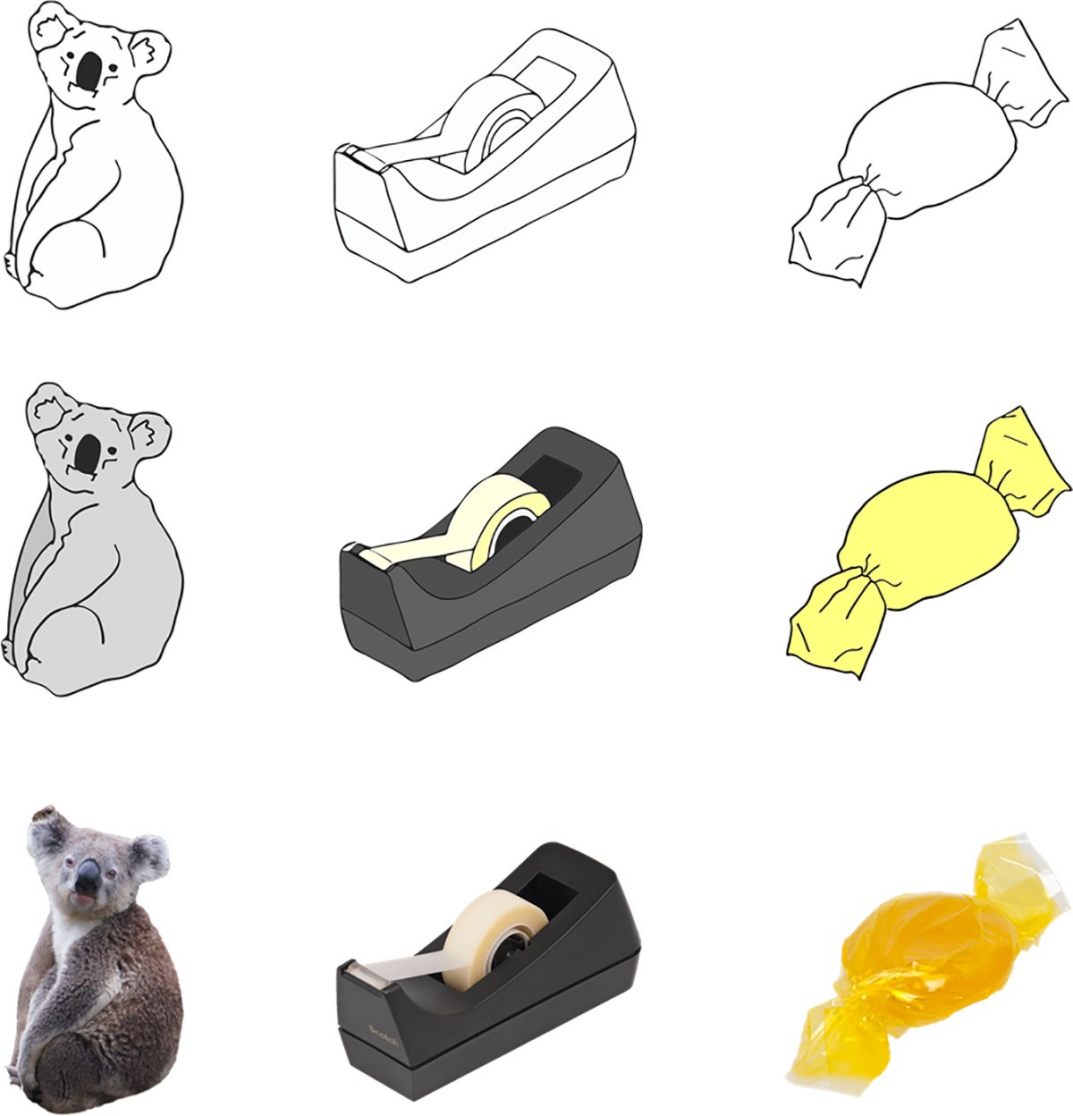
Examples of pictures of the bank in photograph, colored drawing (non-normative), and line drawings. The photographs were retrieved from https://pixabay.com under a CC0 license. The colored drawings and line drawings were created by the authors. The PicPsy bank also includes a colored drawing version but the norms of the present study are collected only for line drawings and photographs.

PicPsy bank consists of 106 concepts each depicted both as a photograph and as a matched drawing (212 pictures total; see Fig 1 for a sample of the stimuli). The bank has also been reproduced in a colored drawings version but the norms of the present study are collected only for line drawings and photographs.

Concepts were selected considering different psycholinguistic such as lexical frequency and length, and considering subjective variables such as familiarity, imaginability, concreteness and subjective age of acquisition. Specifically, concepts of different lexical frequency were selected (*M* = 135.92, *SD* = 209.58, range = 1.14–1,580.43) according to Martínez & García [58]. Additionally, selected words had high familiarity indexes (*M* = 6.03, *SD* = 0.71, range = 3.56–7.00), high imagination indexes (*M* = 6.05, *SD* = 0.56, range = 4.29–6.85) and high concreteness indexes (*M* = 5.87, *SD* = 0.62, range = 3.74–6.97) according to Duchon, Perea, Sebastián-Gallés, Martí, & Carreiras [59]; and a subjective age of acquisition under 8 years old (*M* = 4.24 years, *SD* = 1.32, range = 1.96–7.52) according to Alonso, Fernández, & Díez [60]. Most of the concepts used in PicPsy (75%) were presented in the study of semantic categories by Goikoetxea [61] for 3rd and 4th grade students, and corresponded to the following 30 semantic categories: animals, atmospheric phenomena, birds, buildings, clothing, feelings, flowers, fruits, furniture, geographical accidents and natural land formations, insects, kitchen utensils, light sources, mammals, media, parts of a house, parts of the human body, reading material, tools, trees, types of boats, types of building material, types of drinks, types of fabrics, types of food, types of plants, types of professions, types of relatives, types of toys, and types of vehicles.

In order to depict concepts, public domain photographs under a Creative Commons CC0 license were downloaded from https://pixabay.com, https://commons.wikimedia.org, and https://www.publicdomainpictures.net. Similar to editing steps employed in Brodeur et al. [44], the image background of the photographs were removed, words or brands were blurred, and images were resized to fit in a frame of 500x500 pixels using CorelDraw (Corel Corp., Ottawa, Canada) and Removebg. In order to improve the representation of certain concepts, arrows were added or multiple photographs were combined (e.g., a boy and a girl could be shown together with the arrow pointing to the girl).

The criteria for the arrangement of the photographs were similar to those of Snodgrass and Vanderwart [3]. The photographs of animals or parts of the body were oriented to the right and to the left in a balanced way. Fine and elongated objects (e.g. a pencil) were inclined with an orientation of 45° and the functional part was placed downwards.

The drawings were created directly from the photographs, once edited. Each of the drawings was done delineating the photographs by hand by one of the authors. Each drawing was then digitized and transformed into a vector image, in order to provide definition in any size. All the line drawings had the same visual characteristics of scale, shape and orientation as the corresponding photographs.

### Procedure

Each participant completed the picture naming tasks in one of the two formats, line drawing or photograph. Seventy-three children (61%) and 48 adults (53%) took part in the assessment in line-drawing format, and the remaining participants in colored photograph format. Participants were evaluated from November 2017 to March 2018.

Most of the participants were tested in class groups by one of the examiners in a quiet room of the school or the university, except for 30 adults, who performed the same taskautonomously through the Internet. In order to ensure that children performed the task by themselves, unaffected by the responses of other children, a school teacher accompanied the experimenter in the session and the classroom desks were separated to leave a space of at least one meter between participants. In addition, the children were asked to perform the task in silence and to raise their hands whenever they had questions.

Although the picture naming tasks are usually performed orally and individually, some previous studies have been carried out with children of similar ages to our sample, who perform the studies in written form and in groups (e.g. [34]). This procedure allows for the quick testing of large groups of children, without altering the class’ and school’s routine. If the tasks are properly organized, group testing can offer an efficient method to collect data at minimal cost and time. For a proper written naming performance, children are required to have minimal spelling skills in order to write words fluently. Therefore, a sample of 3rd and 4th graders was selected since by this age children have developed enough fine motor skills to write fluently and it has been shown that Spanish speakers achieve a high level of spelling proficiency by the end of the second grade [62]. In any case, misspellings were not considered when scoring and analyzing the data.

In the classroom-based form, pictures were projected sequentially on the center of a white screen from a computer. Each stimulus was presented preceded by a fixing point (+) for 500 msec and remained on the screen for about 15,000 msec, long enough for participants to respond. The entire session lasted approximately 45 minutes. The order of presentation of the stimuli was randomly generated, but the order of the presentation was fixed.

All participants performed three tasks. These were a picture naming task, a familiarity rating task and a visual complexity rating task. These task were employed in previous picture naming literature with children [4, 12, 13, 34, 37].

For the picture naming task, participants were instructed to name each picture with the first word that came into their mind by asking “What is this picture?” They were also told that they should not worry about spelling. If they failed to recognize the picture or remember the name for a concept, they were told to specify either “Don’t Know the Name” (DKN), “Don’t Know the Object” (DKO), or “Tip-Of-the-Tongue” (TOT) by writing down the initials if they were unable to retrieve a word from memory. As in the previous literature conducted with children and because these tasks conducted collectively, not individually, no information was collected on whether the TOT responses were TOT negative (i.e., when the intended target word does not correspond to with the word that the participant was thinking of) or TOT positive (when the target word does correspond). For the familiarity task, participants were asked to rate how often they daily interact, hear or think about the concept on a scale from 1(a few times) to 5 (many times). For the visual complexity task, participants were asked to rate how difficult the picture was according to the amount of details of the picture on a scale from 1 (few details) to 5 (many details).

Participants were instructed to use an answer sheet created for this purpose, similar to previous written naming studies (e.g., [29]). The sheet contained 106 numbered lines, one for each stimulus, where participants were asked to enter the name of the concept depicted on each picture. To the right of each line, two scales from 1 to 5 were presented for the assessments of familiarity and visual complexity. Here, the participants had to indicate with an X the chosen value. The scale was adapted in a similar way to other studies with children [4, 11]. Specifically, children were instructed to use 1, 3 and 5 point ratings and these values were accompanied by squares of different sizes (1x1cm, 1x3cm and 1x5 cm, respectively) and by oral and written labels. The same scale was presented to children and adults.

In order to reduce the potential difficulties of children performing the experiment, the tasks were selected and adapted according to the following considerations: First, the selected tasks did not require judging any mental images because of the difficulty of children to understand them (see [35]), excluding variables such as image agreement or image variability. Second, the scale was adapted to facilitate its use by children [11] and participants were asked to use points 1, 3 and 5 for their ratings because children could have difficulties to use the full range of numerical values as adults do [4]. Third, a brief practice with three stimuli that were not present in the bank was presented to each participant before starting the study. At this point, questions were also clarified in order to ensure that participants had understood the instructions and the use of the scales. In addition, children were praised with positive feedback on the effort they made during the session, and with a diploma that was awarded at the end of the session. Finally, the duration and progress of the session was scheduled to prevent fatigue (see [37] for a study that finds no fatigue effect in name agreement with a session of similar duration to the one proposed here).

On the online form, stimuli were presented using Google Forms (Google LLC, CA, USA), with one stimulus per page in order to facilitate presentation until participants responded. Each stimulus was presented followed by an online answer sheet, presented as on the classroom-based form.

### Scoring

Previous to the analysis, basic variants of the same name were combined when they were singular and plural forms, and when the name was written with and without spelling mistakes. For each item, the following measures were analyzed.

#### *H* index

The *H* index statistic was proposed by Snodgrass and Vanderwart [3]for the measurement of name agreement in order to assess the dispersion of names for each picture. *H* is calculated as follows:

$$H=\sum_{i=1}^{k}P_{i}\;{log}_{2}\left(\frac{1}{P_{i}}\right),$$

where *k* refers to the number of different name responses given for each picture excluding the DKN, DKO and TOT responses, and *P*_i_ refers to the proportion of participants that gave a name for each picture. The value of *H* is 0 when all participants give the same name to an image and an increase of this value indicates that a greater number of names were provided, increasing the dispersion of responses.

#### Intended name

The intended name corresponds to the word that the researchers used for the search and selection of the picture and, therefore, it is expected to be the most frequent name among the adult participants. The percentage of the participants that gave the intended name for each picture was calculated.

#### Modal name

The modal name corresponds to the name given by the majority of the sample. The percentage of the participants who gave the modal name for each picture was calculated.

#### Alternative names

Alternative names correspond to all names that differ from the intended name. In order to classify the alternative names, we employed the categories and procedure proposed by O’Sullivan et al. [8]. The three categories were as follows. First, correct names that reflect the concept represented by the picture, such as, for instance, synonyms (e.g., *vehículos* [*vehicles*] for *transporte* [*transport*]). Second, incorrect names that refer to a different concept than the one represented by the image, such as physically similar objects (e.g., *bombilla* [*bulb*] for *gota* [*drop*]). And, third, equivocal names that are not correct nouns in Spanish, such as equivocal or invented words (e.g., *rascauñas* [*nails-scratcher*] for *lima* [*nail file*]). Two native Spanish judges carried out this classification and a third Judge was included in cases where no consensus was reached. The percentage of participants who provided alternative names classified in each of the three categories was calculated.

#### Unknown responses

Unknown responses corresponded to all responses provided by the participants with the initials DKN, DKO and TOT described above. The percentage of participants who provided the unknown responses in each of the three categories was calculated.

#### Familiarity

Familiarity measure corresponds to the average value on the 5-point scale for each picture, with small values indicating low familiarity and large values indicating high familiarity. As in the previous literature [34], the measure of familiarity was taken by asking participants to rate the concept rather than the picture itself.

#### Visual complexity

Visual complexity measure corresponds to the average value on the 5-point scale for each picture, with small values indicating low complexity and large values indicating high complexity.

## Results

Table 1 summarizes the norms of the 106 stimuli for name agreement measures and subjective scales sorted by format and age group. We provide norms for two different picture formats (photographs and line drawings). Note, however, that we did not use an experimental design in order to examine differences between formats, because previous literature has already shown that line drawings and photographs have different characteristics that can affect naming performance in children [9] and in adults [7]. To consult detailed norms for each particular stimulus in line drawing and photograph see the data file.

**Table 1:** Means (standard deviations) and correlations between age groups, by picture format.

	Line drawing			Photograph		
	Child	Adult	*r*	Child	Adult	*r*
Name agreement						
*H* index	0.81 (0.74)	0.59 (0.62)	.782***	0.78 (0.75)	0.57 (0.64)	.769***
Intended Name	78.66 (25.01)	87.09 (16.45)	.831***	78.86 (24.27)	85.36 (19.89)	.777***
Modal Name^a^	81.13 (20.97)	87.29 (15.90)	.798***	81.251 (20.42)	86.11 (18.20)	.721***
Subjective scales						
Familiarity	3.18 (0.62)	3.21 (0.88)	.896***	3.14 (0.54)	3.42 (0.74)	.893***
Visual Complexity	2.99 (0.65)	2.90 (0.64)	.872***	2.59 (0.45)	2.61 (0.45)	.744***
Unknown responses	7.03 (9.83)	0.31 (1.29)	.394***	4.35 (6.84)	1.29 (4.13)	.627***
DKN	2.10 (3.19)	0.16 (0.98)	.468***	1.13 (2.48)	0.48 (1.95)	.646***
DKO	3.15 (5.83)	0.06 (0.36)	.421***	0.95 (2.19)	0.44 (1.96)	.397***
TOT	0.71 (1.35)	0.06 (0.47)	.068	1.53 (3.14)	0.37 (1.25)	.290**
Alternative names						
Equivocal names	1.32 (4.05)	0.50 (1.51)	.714***	1.22 (4.68)	050 (1.58)	.727***
Correct names	7.55 (15.30)	5.87 (10.63)	.884***	8.10 (16.44)	7.12 (15.21)	.862***
Incorrect names	12.47 (19.30)	6.54 (10.66)	.767***	11.82 (18.47)	7.02 (12.34)	.718***

The measures of name agreement and subjective ratings were similar to those of the previous literature carried out with older children and adults, but using only line drawings [34]. For example, our results are similar to those reported in Berman et al. [34] in the percentage of modal name (both between 80–88%), familiarity (both slightly above 3) and visual complexity (both slightly below 3).

It should be noted that participants provided modal names that differed from intended names. In line drawings, children named 12 concepts (11%) and adults named two concepts (2%) differently from intended names. Of those, children´s modal names were mostly incorrect names (physically similar such as *cabra* [goat] for *ñu* [wildebeest]) while adults´ modal names were all correct (mostly synonyms) except for one which was incorrect (*reina* [queen] for *princesa* [princess]). In photographs, children named 11 concepts (10%) and adults named six concepts (6%) differently from intended names. Of those, children´s modal names were, in a balanced way, correct names (e.g., synonyms such as *tarro* [jar] for *bote* [pot], or unspecific such as *árbol* [tree] for *pino* [pine-tree]) and incorrect names (e.g., coordinates such as *carta* [letter] for *sobre* [envelope], and physically similar such as *tela* [cloth] for *papel* [paper]). Adult´s modal names were correct names except for two, which were incorrect (*reina* [queen] for *princesa* [princess] and *niños* [children] instead of *fila* [line]).

Name agreement measures, subjective scales, and alternative name responses were strongly correlated between children and adults. In contrast, the correlation coefficients were moderates for DKN, weak for DKO and even non-significant for TOT.

In order to compare the picture naming measures between age groups, we carried out *t*-tests for independent samples (children, adults) separated for line drawings and for photographs.

**Fig 2:**
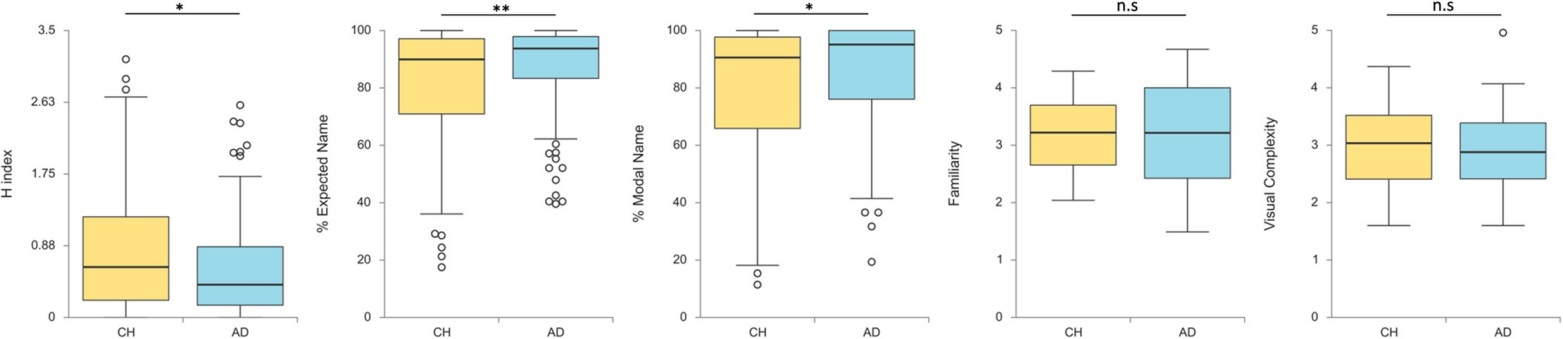
Distribution of line-drawing naming measures by age groups. CH = Children, AD = Adults.

For line drawings, Fig 2 shows box plots for each picture naming variable and differences in children and adults. In line drawings, the results showed significant differences between age groups in *H* index, *t*(210) = 2.36, *p* = .019, *d* = 0.32, percentage of intended name, *t*(210) = -2.90, *p* = .004, *d* = -0.40, and percentage of modal name, *t*(210) = -2.41, *p* = .017, *d* = -0.33. This indicates that adults reached higher name agreement than children. For the subjective scales measures, analyses showed no significant differences between age groups either for familiarity, *t*(210) = -0.34, *p* = .735, *d* = -0.05, or for visual complexity, *t*(210) = 1.08, *p* = .282, *d* = 0.15. This suggests that children and adults provide equivalent rates for the familiarity and visual complexity of line drawings.

In addition, the percentage of unknown responses and the percentage of alternative names in each category were calculated. The results revealed a significant difference between age groups in the percentage of total unknown responses, *t*(210) = 6.98, *p* < .001, *d* = 0.96, as well as in the percentage of unknown categories examined (i.e., the DKN responses), *t*(210) = 5.98, *p* < .001, *d* = 0.82, the DKO responses, *t*(210) = 5.44, *p* < .001, *d* = 0.75, and the TOT responses, *t*(210) = 4.68, *p* < .001, *d* = 0.64. These results indicate higher percentage of unknown responses in children than in adults for line drawings. The alternative names results revealed that children provided significantly higher percentage of incorrect alternative names than adults, *t*(210) = 2.77, *p* = .006, *d* = 0.38, and no significant age group differences were observed for equivocal names, *t*(210) = 1.96, *p* = .051, *d* = 0.27, or for correct names, *t*(210) = 0.92, *p* = .357, *d* = 0.13.

**Fig 3:**
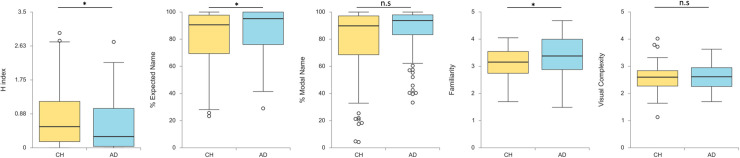
Distribution of photograph naming measures by age groups. CH = Children, AD = Adults.

For photographs, Fig 3 shows box plots for each picture naming variable and differences in children and adults. As in line drawings, a small but significant age group difference was found in *H* index, *t*(210) = 2.11, *p* = .036, *d* = 0.29, and in the percentage of intended name, *t*(210) = -2.13, *p* = .034, *d* = -0.29, but it did not reach significance concerning the percentage of modal name, *t*(210) = -1.83, *p* = .069, *d* = -0.25. Adults showed less dispersion of responses and higher percentage of intended name responses of photographs than children. However, the percentage of modal names was equivalent in both age groups. Furthermore, analyses of subjective scales displayed lower rates of familiarity in children than in adults, *t*(210) = -3.20, *p* = .002, *d* = -0.44, and equivalent rates of visual complexity, *t*(210) = -0.33, *p* = .741, *d* = -0.04.

We also found higher percentages of total unknown responses in children as compared to adults, *t*(210) = 3.94, *p* < .001, *d* = 0.54, the DKN responses, *t*(210) = 2.12, *p* = .035, *d* = 0.29, the DKO responses, *t*(210) = 1.78, *p* = .077, *d* = 0.24, and the TOT responses, *t*(210) = 3.55, *p* < .001, *d* = 0.49, but these differences were from medium to small size. As in alternative names of line drawings, the only significant difference was observed in the incorrect alternative responses, *t*(210) = 2.22, *p* = .027, *d* = 0.31, with equivalent percentage of equivocal, *t*(210) = 1.50, *p* = .135, *d* = 0.21, and correct, *t*(210) = 0.45, *p* = .655, *d* = 0.06, alternative names for both age groups.

## Discussion

In response to the first purpose of this research, we have presented here PicPsy, a new free standardized bank that includes line drawings and photograph stimuli with norms for several relevant psycholinguistic variables using a sample of children and adults. The number of name agreement measures studied for each stimulus (including *H* index, percentage of modal name responses, percentage of intended name responses, percentage of unknown responses, and percentage of alternative names) contributes to a better description of the standardized bank.

According to our second purpose, that was to compare several norms in picture naming between children and adults, we found statistically significant age group differences in all variables examined except for visual complexity and familiarity (only in line drawings). Some of these differences were small, and correlations between both samples were high, but nevertheless it should be noted that, when working with children, some significant differences may appear. These differences can be summarized as more dispersion of names, higher percentage of the intended name, higher percentage of incorrect alternative names, and particularly, higher percentage of unknown responses provided by children as compared to adults in both picture formats. These results concur with previous studies that compared line-drawing naming of same stimuli in children and adults in different languages. Data varied across studies because the stimuli set and samples´ age were different, but all of the studies we are aware described higher values of *H* index and lower percentage of modal names for children than for adults [4, 13, 37]. The present research completes these results analyzing also errors in alternative responses and omissions, and extends the findings to photograph stimuli. Moreover, subjective scales of familiarity (only in line drawing) and visual complexity were rated similarly by both age groups, as in previous literature [4, 13, 34]. Interestingly, photographs were rated as significantly more familiar by adults than by children. A possible explanation may lie in the frequency with which children and adults are exposed to different types of images. While pictures often appear in products and materials designed for children (e.g., children´s literature and cartoons), photographs are common in most products used in secondary education (e.g., textbooks) through adulthood (e.g., newspapers). Thus, throughout their lives, adults have been exposed to a larger number of photographs than children have. However, the difference in exposure to drawings may not be large between children and adults, because exposure to drawings occurs mostly during childhood. In this vein, adults may judge the words represented by the photographs more familiar than children may.

The amount of variables compared here using the same stimuli bank for both age groups and the inclusion of two picture formats allows us to advance some interesting conclusions. Previous literature had already revealed high correlations between both age groups and some authors had pointed out that age differences might be trivial [34]. However, other authors have pointed out that measures relying on modal names ignore children´s errors and sometimes omissions, which are critical in selecting stimuli for studies with children [38]. Our results showed correlated but significantly different name agreement for children and adults. Indeed, the differences in name agreement indicated that children reached lower name agreement than adults. In particular, the larger difference was found in the percentage of unknown responses with weak correlations between children and adults. Additionally, children differed from adults in the quality of alternative responses, providing mostly incorrect alternative names, and approximately twice as much as those given by adults. In line with Cannard et al. [38], our results suggested that the classical measures of name agreement, when complemented by the analysis of unknown and alternative responses, may enrich ​​the utility and quality of a stimulus bank for a given population. In the case of children compared to adults, our findings revealed that we must address omissions, as they may reflect a lack of knowledge for some of the concepts [4], and inaccuracy of meaning of the concept represented by the picture [8] more than perceptual or functional differences between age groups.

We will discuss bellow several contributions of this normative dataset that might be highlighted as compared to previous studies. First, to the best of our knowledge, these are the only picture naming norms for Castilian Spanish-speaking children. Second, the line-drawings set we provide in this study was vectorized, thereby allowing high quality in any size and defined contour. Third, we provide the first standardized bank that includes photograph stimuli with norms using a sample of children. Fourth, the picture bank is freely available with a public license description. Five, we provide several name agreement measures, including an analysis of the percentage of omissions and the alternative names classified within O’Sullivan et al. [8] categories, which, to our knowledge, have not been reported in the previous literature in age groups comparisons. Therefore, the pictures and data presented here, from a sample of children and adults, include a bank of photographs and vectorized line drawings, freely available. The bank is also available for download in a colored drawing version but it is important to remember that the norms of the present study are collected only for line drawings and photographs. For all of the above, we believe that PicPsy is a useful tool for researchers and teachers of Spanish-speaking children. In research and assessment with children, the use of pictures allows the exclusion of possible effects derived from reading skills; and in education, both line drawings and photographs are useful stimuli because part of the vocabulary learning process is linked to the exposure to a rich variety of pictures [63, 64].

Some limitations remain in the current study. In this work, we aimed to present norms of picture naming in children with a sample of at least 100 participants, which is unusual in the literature. However, the children sample is still relatively small (N = 118). Therefore, the data reported here should be taken as preliminary norms for children aged 7–10 years, and it would be desirable to extend the sample and make it stratified in a wider age range. In particular, it would be advisable to collect norms for younger children, considering that previous research had shown that children under the age of 8 are less efficient than older children and adults in picture naming [4, 38], which could lead to more significant differences between age groups or in different variables than those observed herein. In addition, pre-readers would be of great interest, because images, unlike words, are often the only option available for presenting the stimuli to them when conducting research. It should also be noted that the procedure for this study involved group testing and a written naming task. Although this procedure offers an efficient method with significant time and cost savings, it may differ from the results of other experiments conducted individually or orally. Research carried out with adults suggests that spoken and written naming yield similar results [27, 57] and normative studies have been conducted on either of the two naming procedures, but it would be interesting for future studies to experimentally examine potential differences between spoken and written picture naming in children. Finally, it could be interesting to include other measures such as naming speed to test age group differences in naming retrieval in both picture formats.

## Conclusions

In conclusion, the current research offers the first bank of photographs and line-drawings with norms for children, and the first picture naming norms for Castilian Spanish-speaking children. In addition, our results comparing the performance of children and adults indicated differences between them in most of the picture naming variables studied. These results suggest that researchers should take into account the specificities of this population by providing appropriate pictorial stimuli for children. This study has implications for the selection of picture banks and we believe that future researchers and teachers could benefit from using PicPsy for a wide range of psychoeducational programs and tasks.
